# Determination of artemether and lumefantrine in anti-malarial fixed-dose combination tablets by microemulsion electrokinetic chromatography with short-end injection procedure

**DOI:** 10.1186/1475-2875-12-202

**Published:** 2013-06-13

**Authors:** N’Cho Christophe Amin, Huguette Fabre, Marie-Dominique Blanchin, Jérôme Montels, Michèle Aké

**Affiliations:** 1 Département de Chimie Analytique, Bromatologie, Chimie Minérale et Chimie Générale, UFR Sciences Pharmaceutiques et Biologiques - Université Félix Houphouët-Boigny, Abidjan BP V 34, Côte d’Ivoire; 2 Laboratoire de Chimie Analytique, Contrôle physico-chimique des médicaments, Institut des Biomolécules Max Mousseron, UMR 5247, UFR Pharmacie, Université Montpellier 1, Montpellier BP 14491-34093, France

**Keywords:** Antimalarial, Artemether, Lumefantrine, MEEKC, Short-end injection procedure

## Abstract

**Background:**

Artemether-lumefantrine (AL) combination therapy is now the most used anti-malarial treatment in the world. Quality control of AL formulations is still a major challenge in developing countries. Until now, only liquid chromatographic methods have been reported in the literature for their analysis. Capillary electrophoretic methods, which present various advantages (low price of capillary, low volumes of electrolyte consumption), may be an alternative to liquid chromatography methods. In this paper, a reliable method was developed and validated for the determination of AL in commercial fixed-dose combination tablets commercialized in Côte d’Ivoire.

**Methods:**

Artemether and lumefantrine were determined by microemulsion electrokinetic chromatography using short-end injection procedure. The two analytes were extracted from tablets by acidified methanol. Pyrimethamine was used as internal standard. Separation was carried out in an uncoated fused silica capillary, 30 cm long × 50 μm internal diameter, using an effective length of 10 cm and a microemulsion composed of octane, butanol, sodium dodecyl sulfate and borate buffer as background electrolyte, a - 500 V.cm^-1^ electric field and a detection wavelength of 214 nm.

**Results:**

Artemether, lumefantrine and pyrimethamine were separated in 6 min. The method was reliable with respect to selectivity towards formulation excipients, linearity of the response function (r^2^ > 0.998), recovery studies from synthetic tablets (in the range 99–101%), repeatability (relative standard deviation 1–3%, n = 7 analytical procedures). Application to four commercial formulations containing 20/120 mg of AL per tablet gave a content in good agreement with the declared content. However, the electropherogram of one tablet formulation showed the presence of an ingredient which was not declared.

**Conclusion:**

The developed MEEKC method can be proposed as an alternative method to liquid chromatography for the determination of artemether and lumefantrine in fixed-dose combination tablet formulations.

## Background

**Figure 1:**
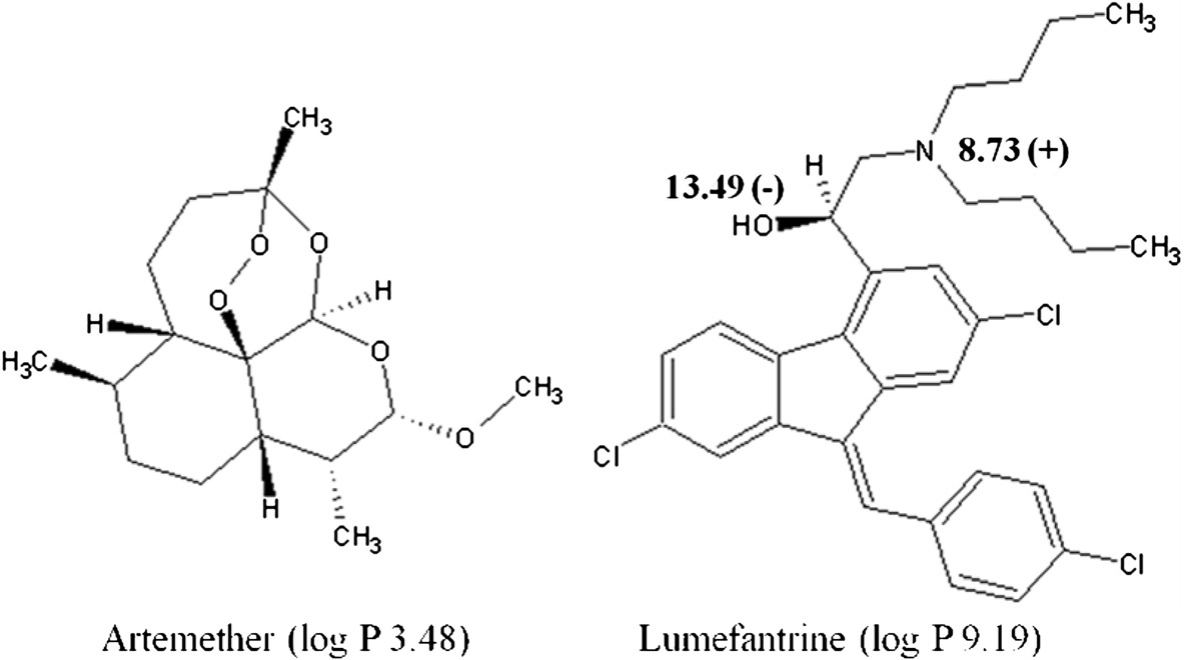
Chemical structure of artemether and lumefantrine with indication of pKa and log P values.

**Figure 2:**
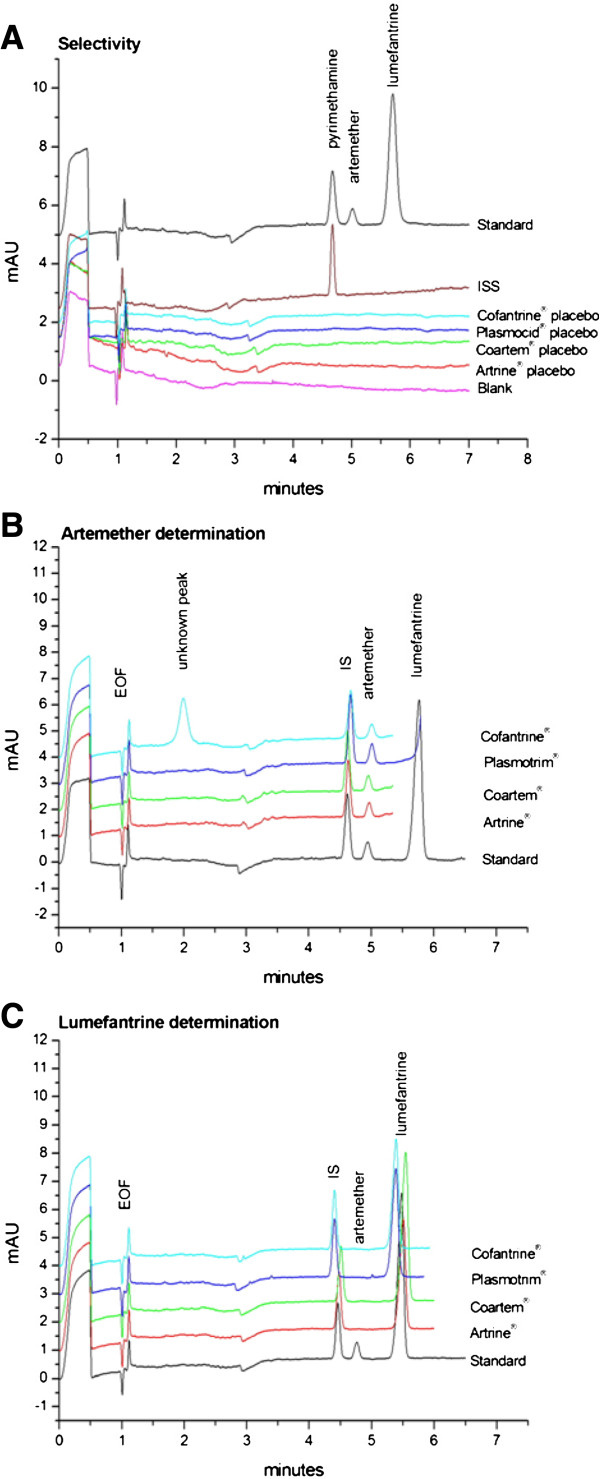
Selectivity towards declared formulation excipients (A) and electropherograms of artemether (B) and lumefantrine (C) determinations.

Malaria is the most important infectious disease in the world, with an estimated 274 million more cases and 1.1 million more deaths between 2001 and 2010 [1]. To improve therapeutic efficacy and delay the development of resistance, the World Health Organization has since 2001 recommended for antimalarial treatment, the use of combination therapy based on the synergistic or additive potential of two or more drugs. Artemisinin-based combination therapy, using artemether-lumefantrine (AL) (Figure 1, [2,3]) and artesunate-amodiaquine (AS-AQ), is currently considered as the first choice treatment for *Plasmodium falciparum* malaria in endemic areas. Tablets and capsules are used in fixed-dose combinations (FDCs) which ensure that the two drugs are taken together and in correct proportions.

The assay of the active substances in these formulations is difficult due to the polarity difference between the analytes and the absence of chromophore for artemisinin derivatives (artesunate or artemether) present in low proportion with respect to the associated anti-malarial drug (weight ratios of 1/2.7 for AS/AQ and 1/6 for AL). For the assay of AS and AQ in FDCs, high performance liquid chromatography (HPLC) [4-6] and capillary electrophoresis (CE) methods have been proposed [7]. For AL, only HPLC methods [8-12] have been reported. César *et al.*[8] were the first authors to propose a HPLC method allowing a separation of the two analytes (within 5 min.) using a cyano stationary phase, an acetonitrile-0.05% trifluoroacetic acidin water (60:40, v/v) mobile phase and a 210 nm detection wavelength. Due to the high lipophilicity of lumefantrine, a mixture of chloroform and acetonitrile was used to ensure the complete extraction of lumefantrine from tablets. Standard addition method was used for artemether quantitation to compensate its low absorbance in the extract. Two monographs were published in 2009 and 2010 as United States Pharmacopoeia SALMOUS standard for AL capsules [9] and in the International Pharmacopoeia for AL tablets [10]. In both monographs, an identical gradient ion-pairing method with detection at two wavelengths (210 nm and 380 nm for artemether and lumefantrine, respectively) is proposed. Test solution (0.2 g L^-1^ for artemether and 1.2 g L^-1^ for lumefantrine) is prepared by extracting the active substances with a complex diluent composed of ion-pair reagent, water, propanol and acetonitrile. Under the chromatographic conditions used, artemether and lumefantrine are eluted at 19 min and 34 min. respectively and the run time (RT) including the time for column re-equilibration is about 55 min. In 2011, some authors [11], stating that no HPLC method had been previously reported, have developed an isocratic method providing the separation in 12 min. using an ODS stationary phase, a mobile phase composed of methanol-trifluoroacetic acid- triethylamine buffer (80:20, v/v) adjusted to pH 2.8 and a wavelength detection of 210 nm. This paper is quite confusing concerning the quantitation since a test solution (0.8 g L^-1^ for artemether and 4.8 g L^-1^ in acidified methanol) which is out of the calibration range and has a concentration different from the standard solution (0.2 g L^-1^ artemether and 1 g L^-1^ lumefantrine) is used. Very recently Suleman *et al*. [12] have developed and validated a stability indicating assay for the simultaneous determination of AL tablets. After extraction by tetrahydrofuran, artemether and lumefantrine are separated in isocratic conditions using a fused-core amide stationary phase and detection at 210 nm (β- artemether) and 335 nm (lumefantrine).

A recent exhaustive literature survey on CE methods applied to anti-malarials [13] has shown that up to date, there are no CE methods reported for the assay of artemether and/or lumefantrine as drug substances or in drug formulations. Since CE presents the distinct advantage of reduced operating cost and low-cost CE instruments have been implemented in several developing countries [14,15] for the quality control of drugs and detection of counterfeit or substandard formulations, the possibility of using this technique for the assay of AL in FDCs formulations is investigated in this paper.

## Methods

### Chemicals

De-ionized water doubly distilled was used throughout the study. All chemicals were of analytical grade. Pyrimethamine used as internal standard (IS), phosphoric acid, sodium dodecyl sulfate (SDS) and 1-butanol were purchased from Sigma Aldrich (Saint Quentin Fallavier, France). Lithium dodecylsulfate (LiDS) was from Fisher Scientific (New Jersey, United States of America), octane and *di*-sodium tetraborate decahydrate from Merck (Darmstadt, Germany). Artemether and lumefantrine were obtained from Quimdis (Levallois-Perret, France).

Pharmaceutical formulations, Artrine® (LIC Pharma, Abidjan, Côte d’Ivoire), Coartem® (Novartis Pharm, Basel, Switzerland), Cofantrine® (EGR Pharma, Maharashtra, Inde) and Plasmocid® (Cipharm, Abidjan, Côte d’Ivoire) were purchased in Côte d’Ivoire. All formulations have a declared content of 20 mg of artemether and 120 mg of lumefantrine per tablet.

### Solutions

#### Background electrolyte solution

The microemulsion (ME) was prepared by accurately weighing 0.81 g octane, 6.61 g 1-butanol, 3.31 g SDS and 89.27 g of 10 mM sodium tetraborate buffer in a 100 mL flask. The mixture was sonicated for 20 min to form an optically transparent ME which is stable for at least three months.

#### Internal standard solution

The internal standard solution (ISS) was a 200 mg L^-1^ pyrimethamine solution in methanol – water – phosphoric acid (93: 5: 2, v/v/v).

#### Mixed standard solution of artemether and lumefantrine

Approximately 20 mg lumefantrine and 200 mg artemether accurately weighed, were transferred into a 20 mL glass volumetric flask, sonicated and diluted to volume with ISS. This solution diluted 1/10 (v/v) in the ME was the standard solution (100 mg L^-1^ lumefantrine, 1000 mg L^-1^ artemether, 20 mg L^-1^ IS).

#### Lumefantrine test solution

A portion of ten tablets finely powdered, equivalent to about 10 mg lumefantrine, was transferred into a 10-mL volumetric flask, sonicated for 20 min. and diluted to volume with ISS. The supernatant obtained after centrifugation (10,000 rpm for 5 min.) and diluted 1/10 (v/v) in the ME was the test solution for lumefantrine (theoretical concentration 100 mg L^-1^ lumefantrine, 20 mg L^-1^ IS).

#### Artemether test solution

A portion of ten tablets finely powdered equivalent to about 10 mg artemether was transferred into a 10 mL volumetric flask, sonicated for 20 min. and diluted to volume with the ISS diluted 1/10 (v/v) in ME. The supernatant obtained after centrifugation (10,000 rpm for 5 min.) was the test solution for artemether (theoretical concentration 1000 mg L^-1^ artemether, 20 mg L^-1^ IS). Because of the physical instability of this microemulsion in the presence of methanol at a concentration higher than 8% v/v [16], standard and test solutions were analysed within six hours following their preparation.

### Apparatus and operating conditions

All experiments were performed on a Beckman P/ACE MDQ (Fullerton, CA) CE instrument equipped with a DAD. All separations were carried out in an uncoated fused-silica capillary, 30 cm long (10 cm to the detector), 50 μm inner diameter, 375 μm outside diameter (Beckman), housed in a cartridge with a 200 × 800 μm detector window. Prior to its first use, the capillary was washed at 20 psi for 20 min. with a 0.1 M sodium hydroxide solution, and then flushed with water for 5 min. On each working day, starting a sequence, the capillary was rinsed at 20 psi for 5 min with 0.1 M sodium hydroxide, 5 min water and 5 min ME.

The optimal operating conditions were as follows: the capillary was flushed with the background electrolyte (BGE) for 2 min. using a rinse vial different from the two separation vials. Sample introduction (~4 nL) was performed on the detector side (short-end injection) by hydrodynamic injection (0.2 psi for 5 s), followed by a “wait” step in another vial containing the electrolyte to prevent sample carryover on the outside capillary tip and contamination of the separation electrolyte. Separation was carried out at 25°C, applying a - 15 kV voltage (− 500 V.cm^-1^) with the UV detector set at 214 nm. Separation vials were changed after 10 injections to take into account buffer depletion.

Standard and test solutions were injected in duplicate. Relative corrected peak areas (RCPA) corresponding to areas/respective migration times (MTs) of analyte/IS were used for calculations.

## Results and discussion

### Method development

The development of a CE method for AL was a rather difficult task since artemether, which does not have chromophore, is in low proportion (weight ratio 1/6 with respect to lumefantrine) in the formulation. Furthermore, lumefantrine is a highly hydrophobic compound (Figure 1). Preliminary experiments were conducted using conventional long-end injection (effective length, 20 cm) and a 15 kV separation voltage (normal polarity) to keep the current developed below 100 μA.

#### Selection of operation mode, electrolyte and separation conditions

The chemical structure (Figure 1) of artemether and lumefantrine shows that artemether is a non-ionizable compound, so that only micellar electrokinetic chromatography (MEKC) and MEEKC can be potential operation modes for a quantitative analysis of both compounds. In a previous paper, it was shown [7] that using a SDS-MEKC method (SDS 30 mM in 25 mM borate buffer pH 9.2, 500 V.cm^-1^) lumefantrine was not eluted within 60 min. This was related to its insolubility in the background electrolyte. Addition of an organic modifier (methanol or acetonitrile) to the BGE at different concentrations up to 30% (v/v) to improve lumefantrine solubility could not achieve lumefantrine elution. Hence, further investigations were carried out in MEEKC, since in MEEKC the presence of a co-solvent (typically 1-butanol or 2-propanol) and an oil (typically octane or heptane) results in a great solubilizing power both for water-insoluble and water-soluble compounds [17]. MEEKC is particularly well suited for the analysis of very lipophilic compounds, such as fat-soluble vitamins or steroids [18,19] and has found a large use for the lipophilicity determination of chemical substances [13,20,21].

Several ME systems were investigated (Table 1), which gave a high resolution (> 5) and an acceptable current (< 90 μA) within an acceptable elution time. The ME composed of 0.81% w/w octane– 6.61% w/w 1-butanol– 3.31% w/w SDS– 89.27% w/w 10 mM borate buffer was selected in the further steps of development, as it gives the best compromise in terms of baseline, RT and operating cost by comparison to LiDS. With this ME, the analytes were eluted within 12 min. with an acceptable current and a resolution higher than 7, so that short-end injection technique (sample introduction at the detector side) could be used to reduce the RT. The robustness of this injection mode for quantitative analysis in capillary zone electrophoresis [22-24], MEKC [25], and MEEKC [26] has been reported.

**Table 1:** Microemulsion systems tested

**Composition of the ME**	**Current developed, RT and migration order**
a) 0.81% w/w octane + 6.61% w/w 1-butanol + 3.31% w/w SDS + 89.27% w/w 10 mM borate buffer	+ 70 μA; RT around 12 min; MT_artemether_ < MT_lumefantrine_;
b) 0.81% w/w octane + 6.61% w/w 1-butanol + 3.31% w/w SDS + 89.27% w/w 20 mM borate buffer	+ 85 μA; RT around 15 min; MT_artemether_ < MT_lumefantrine_;
c) 0.81% w/w octane + 6.61% w/w 1-butanol + 3.31% w/w LiDS + 89.27% w/w 10 mM borate buffer	+ 60 μA; RT around 10 min; MT_artemether_ < MT_lumefantrine_;
d) 0.8% w/w octane + 6.6% w/w 1-butanol + 6.0% w/w SDS + 20% w/w isopropanol + 66.6% w/w 25 mM phosphate buffer	+67 μA; RT around 9 min; MT_artemether_ > MT_lumefantrine_; baseline disturbance

Operating conditions: silica capillary, 30 cm (20 cm to the detector); injection 4 nL; separation voltage 15 kV (normal polarity); 25°C; λ = 214 nm.

#### Selection of an extraction diluent and injection solvent for artemether and lumefantrine

The choice of an injection solvent has a significant impact on separation efficiency in MEEKC as in MEKC, specially for lipophilic compounds [27]. Selection of an appropriate solvent to extract artemether and lumefantrine from tablets was a difficult challenge since they have very different polarities. In MEEKC, it is recommended whenever possible to dissolve the sample in the MEEKC buffer [28] to avoid baseline disturbance. However, lumefantrine is not soluble in the microemulsion but soluble in methanol acidified with phosphoric [11] or formic acid [29]. Because the use of an organic solvent results in a loss of efficiency as it disrupts the microemulsion environment adjacent to the injection zone, the methanolic extract was diluted (1/10, v/v), in the ME prior injection. In addition, because there is no stacking effect, low injection volumes were used to avoid peak broadening. Different injection volumes (from about 1.6 to 12 nL) were investigated. A volume of about 4 nL was found to give satisfactory peak shapes and reasonable signals.

#### Selection of an internal standard

An IS was needed to take into account possible methanol evaporation during the extraction step, small variations in the injected volume. In addition, it takes into account variations due to temperature and electroosmotic flow (EOF) rate. Benzoic acid, sorbic acid, cinchonine and pyrimethamine were tested as IS. Pyrimethamine was selected as it gives a resolution > 2 with artemether and do not increase the analysis time.

#### Capillary rinse between injections

Different capillary rinse procedures between injections were investigated. It was found that a short capillary rinse with the microemulsion was sufficient to obtain acceptable drift and repeatability of MTs (n = 6; relative standard deviation (RSD) < 1%). No fouling of the capillary was observed within 50 injections.

#### Selection of detection wavelength

Due to the lack of chromophore and the need of a sensitive detection for artemether, a 214 nm wavelength was selected for the determination of both analytes as it gives the best signal-to-noise ratio for artemether. Specimen electropherograms of a standard solution and test solutions recorded under the final operating conditions are given in Figure 2.

### Evaluation of method performances

The method was validated according to the international conference of harmonisation guideline [30].

#### Selectivity

The non-interference of the ingredients present in the different commercial tablet formulations analysed (Table 2) was assessed by injecting placebo solutions of each formulation. No interference was noted at the MTs of the active substances and internal standard (Figure 2A).

**Table 2:** Excipients of the commercial formulations analysed

**Excipients**	**Artrine®**	**Coartem®**	**Cofantrine®**	**Plasmocid®**
Colloidal anhydrous silica		x		
Hydroxypropylcellulose				x
Hypromellose		x		x
Isopropyl alcohol			x	
Lactose			x	
Magnesium stearate	x	x	x	x
Methylene chloride			x	
Microcrystalline cellulose	x	x		x
Polysorbate 80 = tween 80		x	x	
Sodium croscarmellose		x		x
Sodium laurylsulfate sodium				x
Starch	x		x	
Talc				x

#### Linearity of the response function

The linearity of the response function (relative corrected peak area (RCPA) analyte/IS) *vs* analyte concentration was assessed by injecting in triplicate mixed standard solutions (in the range 600–1400 mg L^-1^ for artemether and 60–140 mg L^-1^ for lumefantrine) at five concentration levels corresponding to 60, 80, 100, 120 and 140% of the target concentration used in the assay. Corresponding regression equations were:

$$\begin{array}{l} \mathrm{RCPA}\left(\mathrm{artemether}/\mathrm{IS}\right)=\left(0.00038\pm 0.00001\right)\\ \;\mathrm{artemether}\;\mathrm{mg}\;L^{-1}\\ \;–\left(0.03\pm 0.03\right);r^{2}=0.9998 \end{array}$$

$$\begin{array}{l} \mathrm{RCPA}\left(\mathrm{lumefantrine}/\mathrm{IS}\right)=\left(0.0346\pm 0.0005\right)\\ \;\mathrm{lumefantrine}\;\mathrm{mg}\;L^{-1}\\ \;–\left(0.002\pm 0.006\right);r^{2}=0.9987 \end{array}$$

with the confidence intervals calculated at α = 0.05. Analysis of variance showed that the calibration graphs were linear and intersected the origin, showing that a single calibration solution may be used for routine analysis.

#### Recovery studies

The accuracy of the method was assessed by performing recovery experiments on laboratory prepared formulations of Coartem®. Three sets of independent determinations were carried out on placebo powder spiked with known amounts of artemether and lumefantrine corresponding to 80, 100 and 120% of the target concentration. Recoveries of artemether and lumefantrine calculated against a standard solution at the target concentration prepared in duplicate are given in Table 3. Mean recoveries for artemether and lumefantrine are in the range 100-101% and 99-100% respectively.

**Table 3:** **Recovery data for artemether and lumefantrine from laboratory prepared formulations of Coartem***®*

	**Exp. No.**	**% of the target concentration**	**Added amount (mg)**	**Found amount (mg)**	**Recovery (%)**	**Mean recovery ± RSD (%)**
**Artemether**						
	1		7.60	7.73	101.7	
	2	80	7.87	7.65	97.2	100.8 ± 3.2
	3		8.21	8.48	103.3	
	1		9.97	9.78	98.1	
	2	100	10.23	10.75	104.2	100.5 ± 3.3
	3		10.52	10.42	99.0	
	1		11.86	11.91	100.4	
	2	120	12.25	12.43	101.5	100.3 ± 1.2
	3		12.72	12.59	99.0	
**Lumefantrine**						
	1		7.55	7.64	101.2	
	2	80	7.77	7.77	100.0	99.8 ± 1.4
	3		7.85	7.72	98.3	
	1		10.16	10.28	101.2	
	2	100	10.19	10.01	98.2	99.3 ± 1.7
	3		10.73	10.55	98.3	
	1		11.75	11.48	97.7	
	2	120	11.93	11.85	99.3	98.8 ± 0.9
	3		12.38	12.30	99.4	

#### System and procedure precision

System precision was evaluated throughout the study. Similar MTs, relative MTs and RCPA were obtained in different circumstances (capillaries from different suppliers, analysis on different days, preparations of different microemulsions). In all cases, the RSD of corrected peak areas was better than 3% (n = 6 injections). These results confirm the robustness of this microemulsion previously reported in the literature [16].

The repeatability of the entire analytical procedure was evaluated by performing seven replicate determinations of artemether and lumefrantrine in commercial Coartem® tablets. The repeatability expressed as the RSD was 1.8% for artemether and 3% for lumefrantrine.

#### Limit of detection and quantitation

The limits of detection (signal-to-noise ratio of 3) and quantification (signal-to-noise ratio of 10) were evaluated from standard solutions of artemether and lumefantrine. Limits of detection were about 164 mg L^-1^ for artemether and 3 mg L^-1^ for lumefantrine, which correspond to 4 mg of artemether and 3.5 mg of lumefantrine per tablet.

Limits of quantitation were 548 mg L^-1^ for artemether and 6 mg L^-1^ for lumefantrine, which correspond to 13.5 mg of artemether and 12 mg of lumefantrine per tablet.

#### Analysis of different tablet formulations

Four commercial tablet formulations were analyzed for active substance content with the proposed method. Duplicate determinations were carried out except for Coartem® (seven determinations). The respective amounts of artemether/lumefantrine were 102.1/98.6, 101.4/98.1, 99.2/96.0 and 96.7/101.2 percent of the declared content for Artrine®, Coartem®, Cofantrine® and Plasmocid® tablets. The formulations comply with the requirements (90–110% of the label claim) of the International Pharmacopoeia. The corresponding electropherograms are presented in Figure 2B and 2C. An unknown peak is present in the electropherogram of Cofantrine® tablets which does not correspond to a declared excipient. Its presence was confirmed in another batch of Cofantrine® tablet.

## Conclusion

The aim of this study was to test if CE could be used as an alternative technique to liquid chromatography for the assay of fixed-dose combination tablets of AL. We have shown that MEEKC is well suited for this purpose. Satisfactory results were obtained for method validation with respect to selectivity, linearity of the response function, recovery experiments and precision. In comparison with the HPLC methods reported in the literature, the disadvantage of MEEKC method developed is that the quantitation of the active substances requires the preparation of two test solutions which increases the analysis time. However, this method presents several advantages. The total volume of organic solvent used for analyte extraction is dramatically lower (about five times) than that used in LC. Concerning the separation step, CE present the distinct advantage of reduced operating cost in terms of elution solvent (a few milliliters per day) and capillary cost (about 4 US dollars for a 30 cm silica capillary). In contrast, HPLC uses large volumes of mobile phase for column equilibration and elution and expensive chromatographic columns. Hence, in developing countries where financial resources are very limited, CE could be the technique of choice.

## Abbreviations

AL: Artemether-lumefantrine; AQ: Amodiaquine; AS: Artesunate; BGE: Background electrolyte; CE: Capillary electrophoresis; EOF: Electroosmotic flow; FDC: Fixed-dose combination; HPLC: High performance liquid chromatography; IS: Internal standard; ISS: Internal standard solution; LiDS: Lithium dodecyl sulftate; ME: Microemulsion; MEKC: Micellar electrokinetic chromatography; MEEKC: Microemulsion electrokinetic chromatography; MT: Migration time; RPCA: Relative corrected peak area; RT: Run time; SDS: Sodium dodecyl sulfate.

## Competing interests

The authors declare that they have no competing interests.

## Authors’ contributions

NCA study design, sample preparation, data collection, analysis and interpretation of data, drafting of manuscript. HF conception of the study, supervision on the progress of the study and revision of the manuscript. M-DB study design and manuscript preparation. JM technical contribution. MA study design, supervision and manuscript preparation. All authors read and approved the final manuscript.

## References

[B1] World Health Organization World malaria report 2012 2012 Geneva, Switzerland: WHO Press

[B2] ACD/Labs v6.0©1994-2002

[B3] Wishart D Borchers C Li L DrugBank Wishart Research Group and the Metabolomics Innovation Centre, http://www.drugbank.ca

[B4] USP-NF USP34 - NF29 2011 Rockville: Twinbrook Parkway

[B5] Arayne M Sultana N Siddiqui F Naseem S Qureshi F Simultaneous determination of pyrimethamine, sulfadoxine, mefloquine, and ibuprofen in pharmaceutical formulations by RP-HPLC Med Chem Res 2010 19 1043 1054 10.1007/s00044-009-9250-4

[B6] Onwujekwe O Kaur H Dike N Shu E Uzochukwu B Hanson K Okoye V Okonkwo P Quality of anti-malarial drugs provided by public and private healthcare providers in south-east Nigeria Malar J 2009 8 22 10.1186/1475-2875-8-22 19208221 PMC2649149

[B7] Amin NC Blanchin M-D Aké M Montels J Fabre H Capillary electrophoresis for the assay of fixed-dose combination tablets of artesunate and amodiaquine Malar J 2012 11 149 10.1186/1475-2875-11-149 22554086 PMC3459704

[B8] César IC Andrade Nogueira FH Antônio Pianetti G Simultaneous determination of artemether and lumefantrine in fixed dose combination tablets by HPLC with UV detection J Pharm Biomed Anal 2008 48 951 954 10.1016/j.jpba.2008.05.022 18602241

[B9] USP’s SALMOUS Standards guideline Lumefantrine and artemether tablets 2009 Twinbrook Parkway, Rockville: The United States Pharmacopeial Convention

[B10] Int P Pharmacopée internationale, 4ème édition 2011 Genève: OMS

[B11] Kalyankar TM Kakde RB Reversed-phase liquid chromatographic method for simultaneous determination of artemether and lumefantrine in pharmaceutical preparation Int J ChemTech Res 2011 3 1722 1727

[B12] Suleman S Vandercruyssen K Wynendaele E D’Hondt M Bracke N Duchateau L Burvenich C Peremans K Spiegeleer BD A rapid stability-indicating, fused-core HPLC method for simultaneous determination of beta-artemether and lumefantrine in anti-malarial fixed dose combination products Malar J 2013 12 145 10.1186/1475-2875-12-145 23631682 PMC3651282

[B13] Amin NC Blanchin M-D Aké M Fabre H Capillary electrophoresis methods for the analysis of antimalarials. Part II. Achiral separative methods J Chromatogr A 2013 1276 1 11 23332780 10.1016/j.chroma.2012.12.024

[B14] Rohrbasser C Rhême D Décastel SR Samuel Montes MLA Veuthey J-L Rudaz S A new capillary electrophoresis device with deep UV detector based on LED technology Chimia 2009 63 890 891 10.2533/chimia.2009.890 28372623

[B15] Taylor P Pharmelp counterfeit drug analysis projet gains pacehttp://www.securingindustry.com/

[B16] Altria KD Clark BJ Mahuzier P-E The effect of operating variables in microemulsion electrokinetic capillary chromatography Chromatographia 2000 52 758 768 10.1007/BF02491002

[B17] Ryan R Donegan S Power J Altria K Advances in the theory and application of MEEKC Electrophoresis 2010 31 755 767 10.1002/elps.200900568 20191538

[B18] Yin C Cao Y Ding S Wang Y Rapid determination of water- and fat-soluble vitamins with microemulsion electrokinetic chromatography J Chromatogr A 2008 1193 172 177 10.1016/j.chroma.2008.04.016 18440539

[B19] Altria KD Application of microemulsion electrokinetic chromatography to the analysis of a wide range of pharmaceuticals and excipients J Chromatogr A 1999 844 371 386 10.1016/S0021-9673(99)00350-7 10636701

[B20] Wong K-S Kenseth J Strasburg R Validation and long-term assessment of an approach for the high throughput determination of lipophilicity (log P_OW_) values using multiplexed, absorbance-based capillary electrophoresis J Pharm Sci 2004 93 916 931 10.1002/jps.20011 14999729

[B21] Wan H Åhman M Holmén AG Relationship between brain tissue partitioning and microemulsion retention factors of CNS drugs J Med Chem 2009 52 1693 1700 10.1021/jm801441s 19256501

[B22] Fabre H Mesplet N Robustness testing for a capillary electrophoresis method using the “short-end injection” technique J Chromatogr A 2000 897 329 338 10.1016/S0021-9673(00)00794-9 11128216

[B23] Blanchin M-D Baalbaki B Bosc N Fabre H Short-end injection technique in capillary electrophoresis for dissolution testing of tablets Anal Chim Acta 2000 415 67 73 10.1016/S0003-2670(00)00852-7

[B24] Altria KD Kelly MA Clark BJ The use of a short-end injection procedure to achieve improved performance in capillary electrophoresis Chromatographia 1996 43 153 158 10.1007/BF02292944

[B25] Andrasi M Bustos R Gaspar A Gomez FA Klekner A Analysis and stability study of temozolomide using capillary electrophoresis J Chromatogr B 2010 878 1801 1808 10.1016/j.jchromb.2010.05.008 20627825

[B26] Furlanetto S Orlandini S Marras AM Mura P Pinzauti S Mixture design in the optimization of a microemulsion system for the electrokinetic chromatographic determination of ketorolac and its impurities: Method development and validation Electrophoresis 2006 27 805 818 10.1002/elps.200500507 16470626

[B27] Sánchez JM Salvadó V Comparison of micellar and microemulsion electrokinetic chromatography for the analysis of water- and fat-soluble vitamins J Chromatogr A 2002 950 241 247 10.1016/S0021-9673(02)00026-2 11990998

[B28] Miola M Snowden M Altria K The use of microemulsion electrokinetic chromatography in pharmaceutical analysis J Pharm Biomed Anal 1998 18 785 797 10.1016/S0731-7085(98)00217-9 9919981

[B29] Huang L Lizak PS Jayewardene AL Marzan F Lee M-NT Aweeka FT A modified method for determination of lumefantrine in human plasma by HPLC-UV and combination of protein precipitation and solid-phase extraction: application to a pharmacokinetic study Anal Chem Insights 2010 5 15 23 20448843 10.4137/aci.s4431 PMC2865164

[B30] International Conference on Harmonisation of Technical Requirements for Registration of Pharmaceuticals for Human use Validation of analytical procedures: Text and methodology 2005 ICH ed., vol Geneva: Topic Q2 (R1)

